# Fucosylated Glycans in α1-Acid Glycoprotein for Monitoring Treatment Outcomes and Prognosis of Cancer Patients

**DOI:** 10.1371/journal.pone.0156277

**Published:** 2016-06-13

**Authors:** Shin Yazawa, Ryo Takahashi, Takehiko Yokobori, Rie Sano, Akira Mogi, Abby R. Saniabadi, Hiroyuki Kuwano, Takayuki Asao

**Affiliations:** 1 Department of General Surgical Science, Gunma University Graduate School of Medicine, Maebashi, Japan; 2 Department of Molecular and Cellular Pharmacology, Gunma University Graduate School of Medicine, Maebashi, Japan; 3 Department of Legal Medicine, Gunma University Graduate School of Medicine, Maebashi, Japan; 4 Department of Pharmacology, Hamamatsu University School of Medicine, Hamamatsu, Japan; 5 Department of Oncology Clinical Development, Gunma University Graduate School of Medicine, Maebashi, Japan; 6 Big Data Center for Integrative Analysis, Gunma University Initiative for Advance Research, Maebashi, Japan; University of Nebraska Medical Center, UNITED STATES

## Abstract

One standard treatment option for advanced-stage cancer is surgical resection of malignant tumors following by adjuvant chemotherapy and chemoradiotherapy. Additionally, neoadjuvant chemotherapy may be applied if required. During the time course of treatments, patients are generally followed by computed tomography (CT) surveillance, and by tumor marker diagnosis. However, currently, early evidence of recurrence and/or metastasis of tumors with a clinically relevant biomarker remains a major therapeutic challenge. In particular, there has been no validated biomarker for predicting treatment outcomes in therapeutic settings. Recently, we have looked at glycoforms of serum α_1_-acid glycoprotein (AGP) by using a crossed affinoimmunoelectrophoresis with two lectins and an anti-AGP antibody. The primary glycan structures of AGP were also analyzed by a mass spectrometer and a novel software in a large number of patients with various cancers. Accordingly, the relative abundance of α1,3fucosylated glycans in AGP (FUCAGP) was found to be significantly high in cancer patients as compared with the healthy controls. Further, strikingly elevated levels of FUCAGP were found in patients with poor prognosis but not in patients with good prognosis. In the current study, levels of FUCAGP in serum samples from various cancer patients were analyzed and 17 patients including 13 who had undergone chemotherapy were followed for several years post operation. FUCAGP level determined diligently by using a mass spectrometer was found to change along with disease prognosis as well as with responses to treatments, in particular, to various chemotherapies. Therefore, FUCAGP levels measured during following-up of the patients after operation appeared to be clinically relevant biomarker of treatment intervention.

## Introduction

The occurrence of tumor-associated alteration of glycan structures in glycoproteins and glycolipids expressed on the tumor cell surface, which leads to the secretion of such molecules with aberrant glycans into the plasma as tumor-associated antigens is widely known [1,2]. Meanwhile, a series of tumor-associated, glycan-based antigens have already been isolated and validated to preferentially predict the presence of tumors in patients, and some have found clinical application as tumor markers, including carcinoembryonic antigen (CEA) [3,4], carbohydrate antigen 19–9 (CA 19–9) [5,6], α-fetoprotein (AFP) [7,8], cancer antigen 125 (CA125) [9,10], sialyl Lewis X-i (SLX-i) [11,12] and sialyl-Tn (STN) [13,14] antigens. These biomarkers reflect progress in past endeavor to detect malignant tumors and follow their prognosis during treatment intervention. However, since the diagnostic powers of these markers are often limited by low sensitivity and high false positive rates, the diagnosis has been conducted in combination with alternative biomarkers to accelerate positive rates and accuracy in practice settings. For example, in pancreatic cancer, a combination assay of serum CEA, CA 19–9 and CA 242 is known to have improved the specificity and sensitivity of diagnosis and prognosis of pancreatic cancer patients [15]. Combined uses of multi tumor markers have also been sought in other cancer patients [16–19]. However, the clinical value of tumor-associated antigens in serum samples seems to be limited as tumor markers in patients with gastric and lung cancers, in particular, when an earlier clinical stage is considered. Further, since the level of such antigens secreted from tumors cells into the plasma are not adequate to reach a positive range [20.21], we have been looking for a suitable and reliable new biomarker.

**Fig 1:**
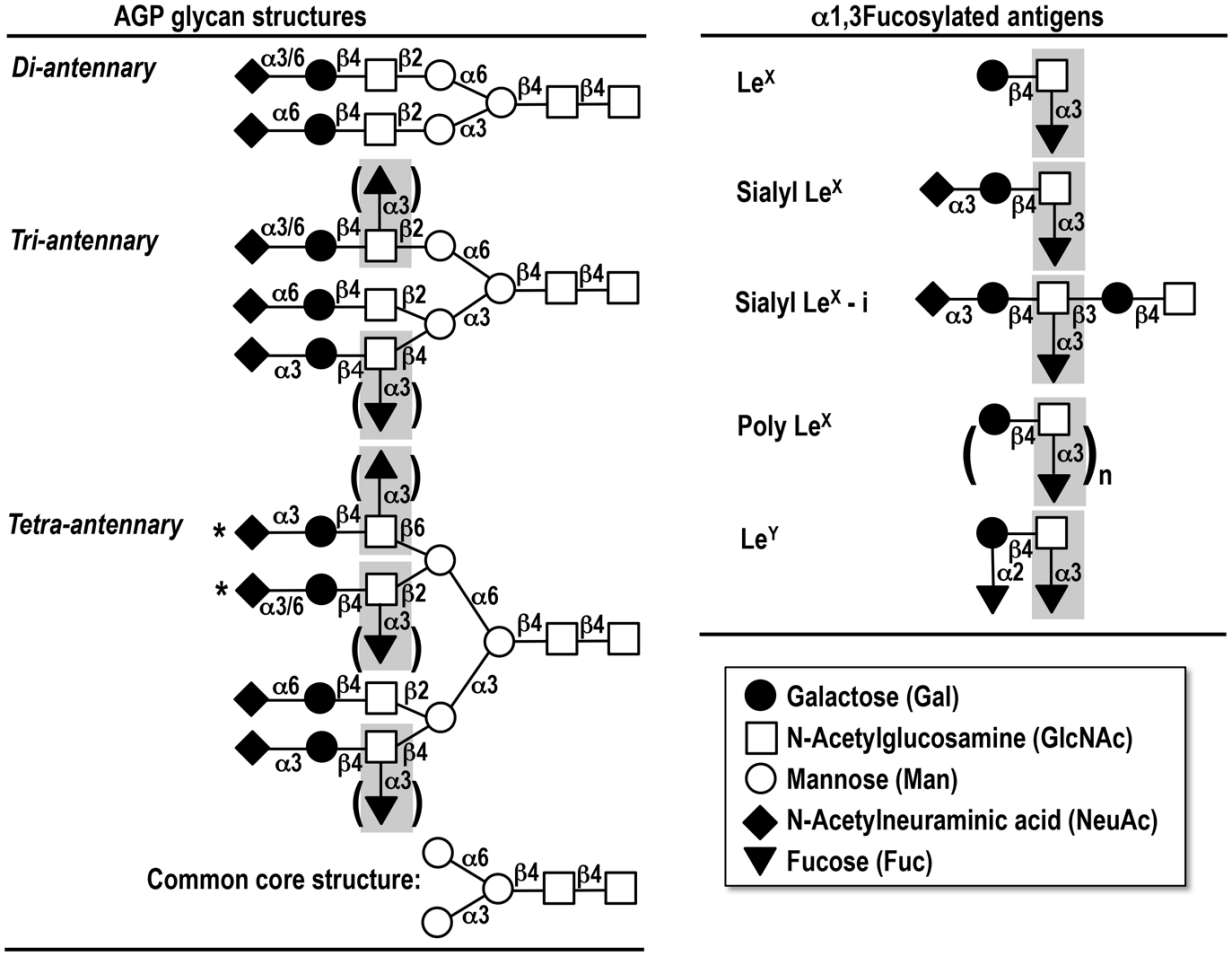
Symbolic representation of α_1_-acid glycoprotein (AGP) glycan structures and the determinants of α1,3fucosylated antigen. One molecule of AGP possesses five glycan chains including diantennary, triantennary and tetraantennary glycans, respectively. All possibilities are shown in brackets and '*'. The common core structure consists of [Man]_3_[GlcNAc]_2_.*Repeated *N*-acetyllactosamine structure [(Galβ1,4GlcNAc)_n_] attached. See the details in Text.

Among the glycosyl linkages found in glycan-based tumor-associated antigens, α1,3fucosyl residues attached to GlcNAc residues are one of the most common structures expressed in tumor cells with very abundant and aberrant existence in plasma and/or serum as LeX (Galβ1,4[Fucα1,3]GlcNAc), sialyl LeX (NeuAcα2,3Galβ1,4[Fucα1,3]GlcNAc), sialyl LeX-i (NeuAcα2,3Galβ1,4[Fucα1,3]GlcNAcβ1,3Galβ1,4GlcNAc), poly LeX ((Galβ1,4[Fucα1,3] GlcNAc)_n_) and LeY (Fucα1,2Galβ1,4[Fucα1,3]GlcNAc) antigens [1, 22–24] (Fig 1).

α_1_-Acid glycoprotein (AGP) is a major plasma glycoprotein with a molecular weight of 41–43 kD and with highly branched *N*-linked glycans, which is synthesized in and secreted from the liver into plasma [25]. The potential physiologic significance of AGP has been investigated as an acute-phase protein, and not only quantitative changes of the molecule but also qualitative changes of its glycan structures have been studied in association with inflammation, pregnancy, estrogen treatment, cancer, liver diseases and autoimmune diseases like rheumatoid arthritis [25–32]. Structural analyses of AGP glycans have revealed that they consist of five complex type *N*-linked glycan chains including diantennary, triantennary and tetraantennary glycans, and sialylation occurs at most of the terminal Gal residues in every four chains. The (sialyl) LeX determinant is present on the lactosamine (Galβ1,4GlcNAc) structures in both tri- and tetraantennary glycans and elongated tetraantennary ones with repeating lactosamine structures as seen in Fig 1 [25,33]. Recently, we investigated AGP glycoforms in a large numbers of serum samples from cancer patients as well as healthy controls by using a crossed affinoimmunoeleoctrophoresis (CAIE) with Con A lectin, *Aleuria aurantia* lectin (AAL) and anti-AGP antibody [34]. Accordingly, patients with advanced malignancies who had AGP glycoforms containing highly branched and fucosylated glycans for long periods after surgical intervention were found to have a poor prognosis. In contrast, patients who had AGP glycoforms without such changes were expected to have a good prognosis irrespective of their clinical stages. In addition, serum AGP levels in preoperative patients were significantly high compared with the level in healthy controls. However, no obvious difference was seen between patients with respect to serum AGP levels and their clinicopathological features including their prognosis after operation. Additionally, in our recent study [35], we developed a novel system for comprehensive analysis of serum AGP glycans with the aid of a mass spectrometer and a homemade software (AGPAS) allowing very rapid determination of primary structures of AGP glycans. It was, then, demonstrated that changes in AGP glycans after operation could indeed be used as a novel biomarker for monitoring and predicting patients’ prognosis. Namely, relative abundance of fucosylated tri- and tetraantennary glycans in AGP molecule (FUCAGP) was found to be significantly higher in cancer patients as compared with healthy controls. Further, highly elevated levels of FUCAGP were found specifically in patients with poor prognosis but not in patients with good prognosis. Since AGP levels were found to respond to changes in clinical events, we also focused on serum AGP levels as well as FUCAGP in the same serum samples from 30 patients with diverse cancers. Among these, 17 patients including 13 patients who underwent chemotherapies were followed for a long period after operation based on their clinicopathological status.

The objective of the current study was to evaluate FUCAGP as a novel glycan-based biomarker to predict clinical outcomes along with patients’ response to medication including various chemotherapies.

## Materials and Methods

### Materials

**Fig 2:**
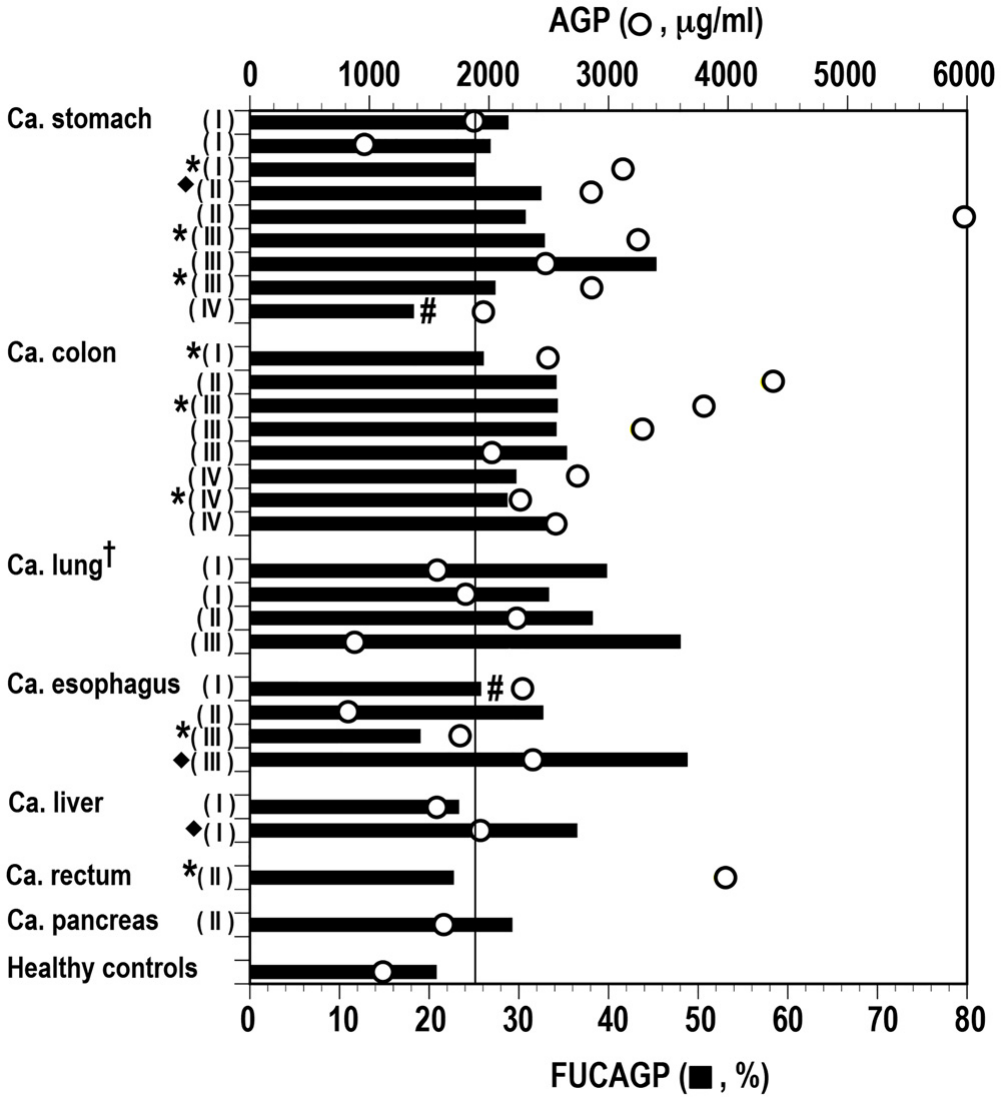
Levels of FUCAGP and AGP concentrations in serum samples from various cancer patients and healthy controls. FUCAGP, relative abundance of fucosylated tri- and tetraantennary glycans in AGP was obtained from MS analysis. AGP concentrations were determined with an ELISA. † One subject (patient N in Fig 3) was excluded. Samples obtained at one (*) and two (◆) postoperative days. #Patients underwent neoadjuvant chemotherapy. Clinical stages of each patient were shown in the parentheses. Vertical bar indicates the cut-off level of FUCAGP set in this study.

Blood samples were obtained from patients with various types of malignancies (n = 30) who were admitted to the Gunma University Hospital (Maebashi, Japan) along with the guideline for informed consent and approval from the Ethics Committee of Gunma University Graduate School of Medicine. This study was approved by the Ethics Committee of Gunma University Graduate School of Medicine, and written informed consent was obtained from all the donors. All clinical stages were determined by the TNM Classification of Malignant Tumours [36]. They consist of patients with gastric (n = 9), colon (n = 8), lung (n = 5), esophagus (n = 4), liver (n = 2), rectum (n = 1) and pancreas (n = 1) cancer (Fig 2). Among them, 17 patients including 13 ones who underwent various chemotherapies were followed up during long pre- and postoperative periods and were explored in detail as described below (Table 1). Blood samples were also obtained from volunteers as healthy controls (n = 21). Serum samples were prepared within a several hours after the blood drawing and were stored at -80°C until use. HiTrap DEAE FF, HiTrap SP FF and HiTrap Desalting were purchased from GE Healthcare (Amersham Place, UK). PNGase F was from Roche Applied Science (Indianapolis, USA). BlotGlyco was obtained from Sumitomo Bakelite, Co. (Tokyo, Japan). Human AGP and 2,5-dihydroxybenzoic acid were purchased from SIGMA (St. Louis, MO, USA). Trypsin, DTT, and other organic agents for BlotGlyco were obtained from Wako Pure Chemical Co. (Tokyo, Japan). Anti-human AGP rabbit serum was obtained from DAKO (Carpinteria, CA, USA) and peroxidase-conjugated anti-human AGP was from Abcam (Cambridge, UK). Tumor-associated antigens in serum samples measured in this study were CEA, cytokelatin 19 fragments (CYFRA), CA 19–9, neuron specific enolase (NSE), squamous cell carcinoma (SCC) and SLX-i. Levels of each antigen were determined by an ELISA using the Cobas system (Roche for CEA, CYFRA, CA 19–9 and NSE), the ARCHITECT system (Abbot for SCC) and by a RIA (Otsuka for SLX-i) following the manufacturer’s instructions. Standard cut-off values of each antigen were set at 5.0 ng/ml for CEA, 2.8ng/ml for CYFRA, 37 U/ml for CA 19–9, 12 ng/ml for NSE, 1.5ng/ml for SCC and 38 U/ml for SLX-i, respectively.

**Table 1:** Prognostic groups of patients followed after surgical operation.

Prognostic group^1^	Patient	Ca.^2^	Stage	Chemotherapy^3^
***Dead***				
Short survival (Chemo/+)				
(died at 231 POD)	A	Stomach	III	Ct. 1, 2, 3
(died at 334 POD)	B	Esophagus	I	Ct. 1, 2, 3
(died at 365 POD)	C	Esophagus	II	Ct. 1, 2, 3
(died at 393 POD)	D	Colon	IV	Ct. 1
(died at 463 POD)	E	Stomach	IV	Ct. 1, 2, 3, 4
Short survival (Chemo/-)				
(died at 257 POD)	F	Lung	III	none
Long survival (Chemo/+)				
(died at 713 POD)	G	Colon	IV	Ct. 1, 2, 3
(died at 860 POD)	H	Stomach	II	Ct. 1, 2, 3
(died at 1,281 POD)	I	Stomach	III	Ct. 1, 2
(died at 1,639 POD)	J	Esophagus	III	Ct. 1, 2, 3, 4
***Alive***				
Long survival (Chemo/+)	K	Stomach	II	Ct. 1, 2, 3
	L	Colon	II	Ct. 1, 2
	M	Esophagus	III	Ct. 1, 2
	N	Lung	I	Ct. 1
Long Survival (Chemo/-)	O	Colon	I	none
	P	Lung	I	none
	Q	Colon	IV	none

^1^ Patients who received chemotherapy (Chemo/+) or no chemotherapy (Chemo/-) treatment and died with a short or long survival after operation at the postoperative day (POD) indicated (***Dead***) or survived during the follow-up periods (***Alive***).

^2^ Ca., carcinoma.

^3^ Ct., chemotherapy with the first line (1) to the fourth line (4) treatment.

### Determination of serum AGP levels

The AGP levels in serum samples were measured by a sandwich-type ELISA using anti-human AGP antibody and horseradish peroxidase-conjugated anti-human AGP as described previously [34]. Results from ELISA of AGP concentrations in healthy control group indicated that the cut-off value was 807μg/ml [34].

### Purification of serum AGP

AGP was purified from 0.5ml of serum samples based on the previous method [35] with a slight modification allowing a rapid and convenient purification procedure. Thus, a newly developed device (AGP-prep-DOCK) consisting of directly connected five columns, namely one HiTrap Desalting, one HiTrap DEAE FF, two HiTrap Desalting and one HiTrap SP columns, was set up. It was flowed with a set of different buffer solutions [35] by means of a programmed pump trough the three-way cock connected to respective columns. By using the AGP-prep-DOCK, purified AGP solution was obtained from the final HiTrap SP column within a short time, and at the same purity as described previously [35]. The purified AGP was used for the preparation of labeled *N*-glycans followed by mass spectrometric analysis.

### Preparation and labeled *N*-glycans of AGP

*N*-Glycans and labeled *N*-glycans of AGP were prepared according to the protocol of the glycosylation kit (BlotGlyco) with a slight modification as described previously [35]. Briefly, samples were digested with trypsin and PNGase F and the resulting *N*-glycans were labeled by means of BlotGlyco. After labeling with the aoWR reagent, an exact ms of each glycan was changed as follows: MW of labeled glycan = [an exact ms]–[H_2_O (18.0105)] + [aoWR (447.223)] + [CH_3_ (14.0156) x n] + [H (1.0078)], where n indicates the numbers of *N*-acetylneuraminic acid residues.

### Mass spectrometric analysis of *N*-glycans of AGP

One μl of the sample solution was mixed with 1μl of the matrix solution (10mg of 2,5-dihydroxybenzoic acid in a mixture of 300μl acetonitrile and 700μl distilled water). Then one μl of the mixed solution was deposited on a MALDI target plate (Ground steel BC, Bruker Daltonik GmbH, Bremen, Germany) and allowed to dry. Mass spectrometric data were obtained using a MALDI-TOF/TOF ultrafleXtreme (Bruker) in reflector, positive ion mode typically summing 20,000 shots. All spectra in the mass range of m/z 1000 to 5000 were obtained and deisotopic masses for each peak picked were exported as a mass peak list with each value of both the centroid mass and the corresponding area to the home-made, operation software, AGPAS recently developed [35].

### Identification of *N*-glycans of AGP

Each glycan molecule was abbreviated by using a four-digit number indicating the number of galactose (Gal), *N*-acetylglucosamine (GlcNAc), fucose (Fuc) and *N*-acetylneuraminic acid (NeuAc) residues consisting of each glycan except the common core structure ([mannose (Man)]_3_[GlcNAc]_2_) (Fig 1). Four hundred and fifty-three theoretically predicted glycans and 154 methylated glycans maximally predicted to be yielded through the labeling process were extracted for constituting the database of the AGPAS [35]. All the primary structures of AGP glycans were therefore assigned rapidly, and in addition, not only the relative abundance of each glycan but also that of fucosylated tri- and tetraantennary glycans (FUCAGP) were simultaneously calculated using their corresponding area. The cut-off value of FUCAGP was arbitrary set at mean + S.D. of healthy controls (= 25.01) as described previously [35]. A better version with some improvement for assignment of glycans in AGPAS was used in this study, and the relative abundance of glycans totally hit was 76.18 ± 8.27% (n = 298).

### Patients and treatments

Serum samples from 30 patients with various malignancies and in different clinical stages seen in Fig 2 were collected before and after surgical interventions. Among these, 17 patients with gastric, esophagus, lung and colon adenocarcinomas were followed for long postoperative days (PODs). The standard adjuvant chemotherapy and various chemotherapies including the neoadjuvant chemotherapy (primary systemic chemotherapy, PSC) were conducted in 13 of the 17 patients according to their individual regimens with up to the fourth line of treatment (Table 1). These 17 patients were classified into 5 subgroups based on their prognostic status as follows. Patients who received chemotherapy (Chemo/+) or no chemotherapy (Chemo/-) treatment and died with a short survival after operation (Short survival); patients who died with a prolonged survival after repeated treatments of chemotherapy (Long survival); patients who underwent chemotherapy, responded well and survived during the follow-up period (Alive, Chemo/+); patients who survived with no chemotherapy treatment during the follow-up period (Alive, Chemo/-).

### Determination of a *FUT6* gene deficient patient

Genomic DNA was extracted from peripheral blood samples by the standard phenol-chloroform extraction method. The *FUT6* gene locus was PCR-amplified with ty-1 and ty-2 primers [37]. Nucleotide sequences of the PCR products were determined by a direct sequence using specific primers for the target.

## Results

### Levels of FUCAGP and AGP in serum samples from cancer patients and healthy controls

*N*-glycans of AGP in serum samples from preoperative patients with various malignancies and in different clinical stages (n = 30) and from healthy controls (n = 21) were analyzed by a mass spectrometer. With the aid of the AGPAS software, relative abundance of each glycan was determined simultaneously and then, relative abundance of fucosylated tri- and tetraantennary glycans in AGP (FUCAGP) was obtained (Fig 2). Relatively low levels of FUCAGP were found in four of the eight patients whose samples were obtained at one day after operation (POD). Further, one of the two patients (patient E in Table 1) who underwent neoadjuvant chemotherapy (PSO) showed the lowest level of FUCAGP (18.17%). However, levels of FUCAGP in cancer patients were still significantly high compared with those in healthy controls (mean ± S.D.: 32.25 ± 6.89 vs. 20.70 ± 6.61; *P* < 0.05). Levels of serum AGP concentration in the same samples were also compared in both patients and healthy controls, and significant elevated levels were found in the patients (mean ± S.D. 2507.33 ± 1093.14. vs. 1119.75 ± 456.65; *P* < 0.05). No correlation was present between FUCAGP and AGP levels both in patients (*r* = 0.117) and healthy controls (*r* = -0.067), respectively.

### Levels of FUCAGP and AGP in cancer patients followed for a long post-operative period

**Fig 3:**
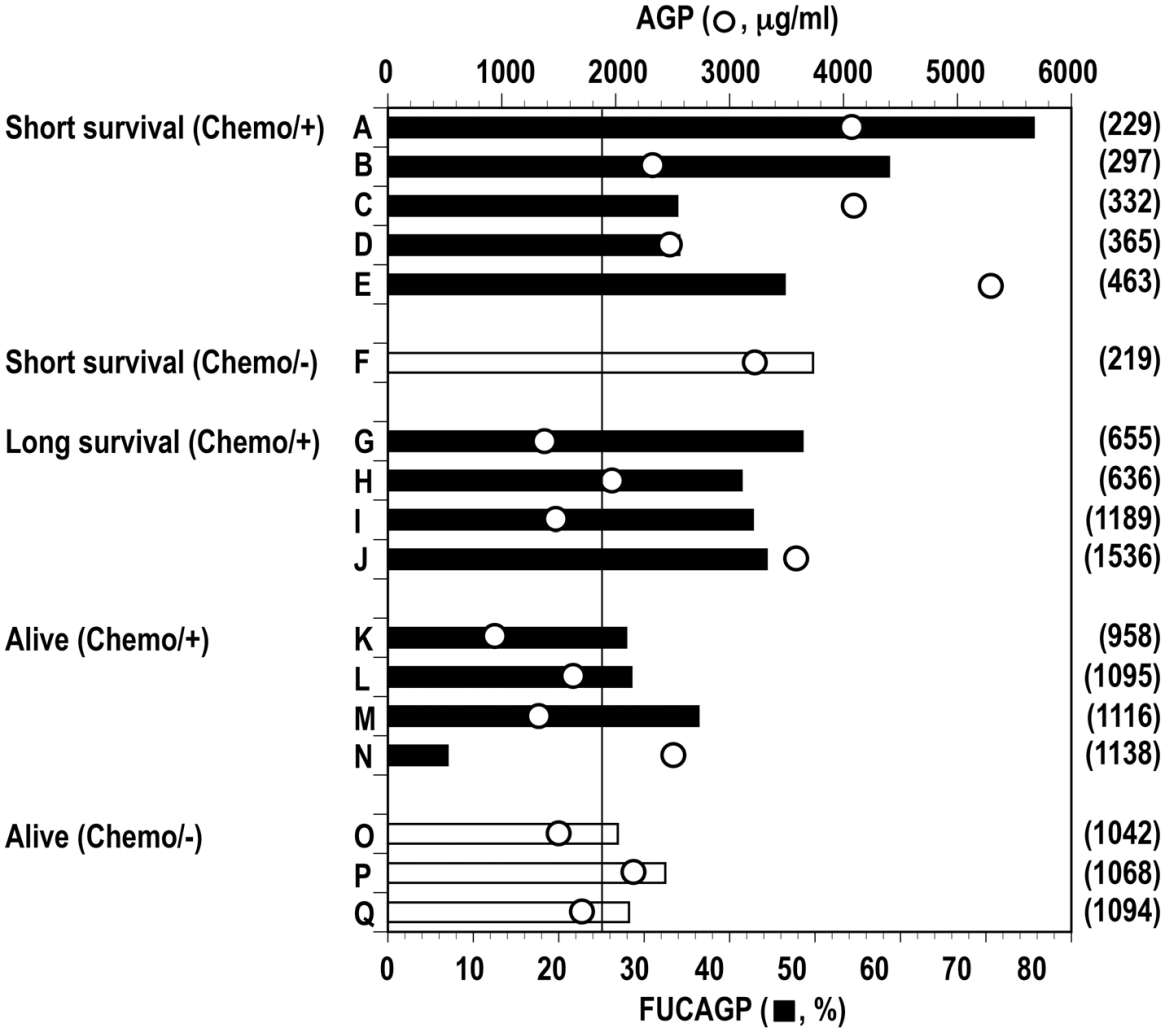
Levels of FUCAGP and AGP concentrations in serum samples from 12 cancer patients followed over long postsurgical periods. Patients (A to Q) were classified according to their prognostic statuses. Refer to Table 1 for the details of prognostic status of each patient. Vertical bar indicates the cut-off level of FUCAGP set in this study. Closed and open bars indicate patients underwent chemotherapy and no chemotherapy treatment, respectively.

Among aforementioned 30 patients, 17 patients classified based on their prognostic statuses (Table 1) were followed for a long postoperative period to analyze their FUCAGP and AGP levels. Thirteen of the 17 patients underwent various chemotherapies according to their individual regimens, and the rest received no such therapies. Patients who died within 463 POD (Short survival) possessed elevated FUCAGP at their latest periods, and strikingly elevated levels of FUCAGP were found in four of the six patients at the period (Fig 3). Further, patients who died with a prolonged survive after repeated treatments of chemotherapy (Long survival) also possessed elevated levels of FUCAGP at their latest periods. However, as seen in patients who had survived with repeated chemotherapies (Alive, Chemo/+) and with no chemotherapy treatment (Alive, Chemo/-), patients with good prognosis showed relatively lower FUCAGP levels at around three years after operation. Altogether, the highest and lowest levels were determined in patients A (75.59%) and N (6.98%), respectively. Whereas, AGP levels were also determined in the same samples from 17 patients. No significant difference of AGP concentrations was found in association with their prognostic statuses, even though elevated levels were commonly observed in cancer patients when compared with those in healthy controls as shown previously [34]. No clear correlation was present between levels of FUCAGP and AGP concentration in these patients.

### Follow-up studies of FUCAGP and AGP levels in cancer patients with a poor prognosis

**Fig 4:**
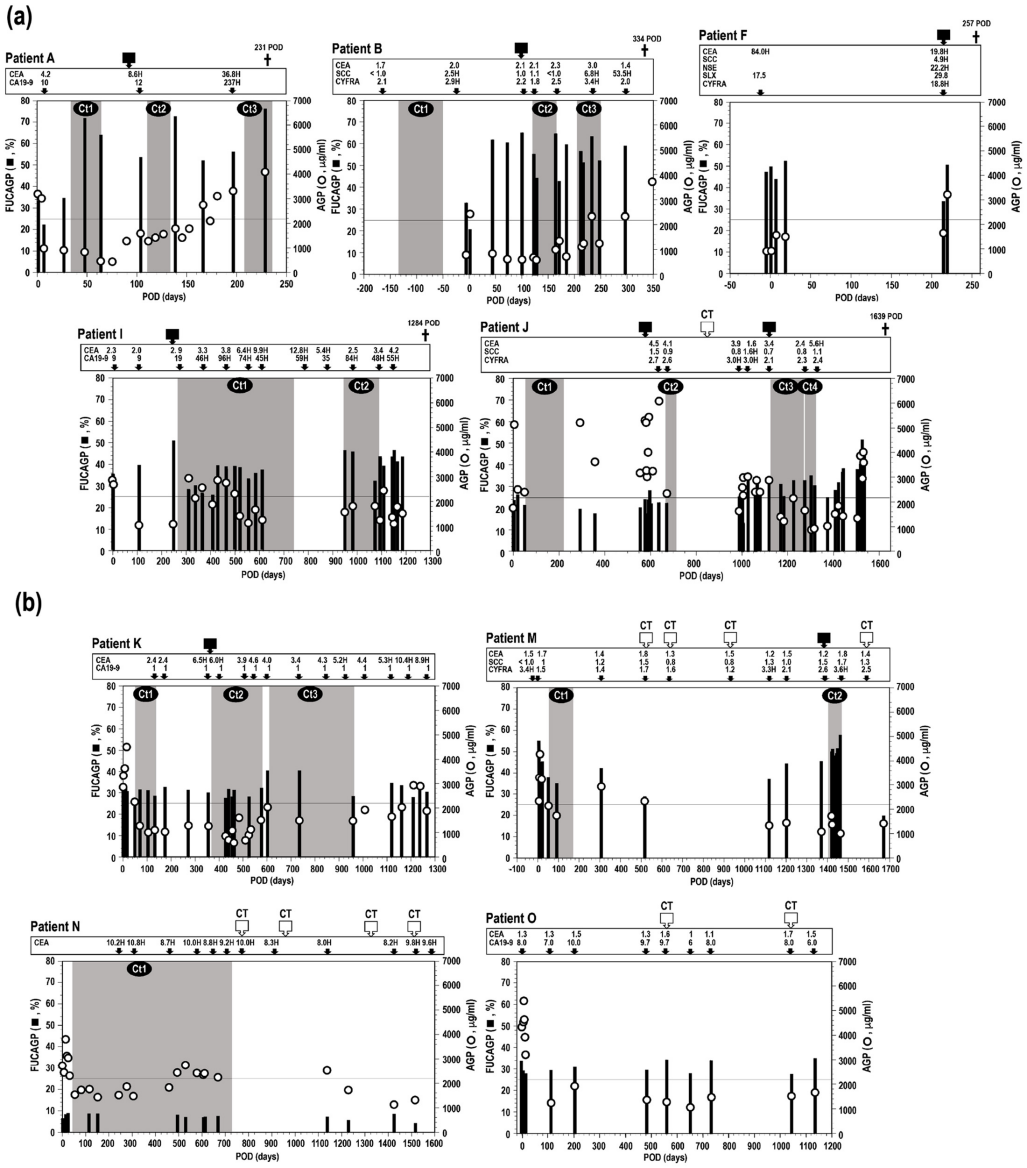
Changes of FUCAGP and AGP levels in serum samples from patients followed for long postsurgical periods. Patients with poor (a) and good (b) prognosis. POD, postoperative days. Solid squares indicate the POD when recurrence and/or metastasis of tumors were clinically determined. Open squares indicate the POD when tumor-free was observed through the computerized tomography (CT) scan. † indicates when the patient died. Each value of tumor markers was measured at the POD indicated with an arrow and numbers with a red-colored letter ‘H’ reveal over the cut-off values indicated in respective instructions. Gray screens on the figure show the duration of chemotherapy with each line of treatment (Ct1—Ct4). Horizontal bars show the cut-off level of FUCAGP set in this study. Refer to Table 1 for the details of prognostic status of each patient.

Patients A and B underwent chemotherapies with up to the third line including the neoadjuvant chemotherapy treated in patient B, but they died within one year after operation. Elevated levels of FUCAGP in these patients dropped below the cut-off level immediately after operation but it was flowed that their levels elevated shortly again and reached to the abnormally high range (Fig 4a). Importantly, the level seemed to be retained during the follow-up period irrespective of repeated treatments of chemotherapies up to the third line, and subsequently they died due to recurrence of diseases. It was of particular interest that such extremely elevated levels of FUCAGP were observed in advance before the recurrence of diseases was detected by means of CT scans or suspected by tumor marker diagnosis. The other patients with a short survival (patients C, D, and E in Table 1) also showed the same change of FUCAGP levels during the observation period indicating that no significant drop of elevated levels of FUCAGP was found even though they received various chemotherapies up to the fourth line and died within 500 POD (data not shown). Highly elevated FUCAGP levels in patient F who had received no adjuvant chemotherapy also shown to be retained before and after operation. The patient who died shortly after the recurrence of disease was detected by CT scans and suspected by diagnosis with four tumor markers.

In contrast, patients I and J with a long survival received repeated chemotherapies for longer periods and survived up to 1284 and 1639 POD, respectively. FUCAGP levels which dropped once after operation but elevated again at 111 POD (patient I) and 1030 POD (patient J) prior to the detection of recurrence by means of CT scans or diagnosis with tumor markers. Interestingly, elevated levels of FUCAGP, then fell around the cut-off level shortly after receiving chemotherapy with the first and third line treatment, respectively against the recurrence. However, the same rising and falling levels of FUCAGP were observed in both patients resulting from the treatment of the next line of chemotherapy and subsequently they died due to the progress of disease accompanying by a continuous elevation of FUCAGP level. The same rising and falling changes of FUCAGP levels were also found at least twice corresponding to each line of chemotherapy in two other patients (patients G and H), but either recurrence and/or metastasis of tumors or no experience of complete remission occurred in these patients and subsequently they died (data not shown). Therefore, patients with a long survival seemed to respond to certain lines of chemotherapy incompletely accompanying by a temporary drop of the FUCAGP level during a few months in the period.

AGP levels in serum samples from patients who died during the follow-up periods were observed to change differently except that remarkable increases were found at the time of operation in most of the patients. No significant correlation was present between AGP concentrations and FUCAGP levels and/or between AGP concentrations and tumor marker levels.

### Follow-up studies of FUCAGP and AGP levels in cancer patients with a good prognosis

Patients K and M had been followed for more than 3 years during they received repeated chemotherapies up to the third line of treatments (Fig 4b). They had relatively low levels of FUCAGP around the cut-off level after operation and adjuvant chemotherapy. Then it was found that FUCAGP levels raised gradually following by detection of the recurrence of diseases or positive results from tumor marker diagnosis. However, after receiving the next line treatment of chemotherapy against the recurrence of diseases, elevated levels of FUCAGP were found to descend correspondingly. It appeared prominently that the highly elevated levels dropped dramatically below the cut-off level in patient M following by negative results obtained from both CT scans and tumor marker diagnosis, and no clinical recurrence was observed during the follow-up period. It was also noteworthy that FUCAGP levels gradually dropped around the cut-off level after detecting recurrence of disease and the second and third line chemotherapy treatments in tumor-bearing patient K, even though positive results of CEA came up during the latest period. It remains to be seen whether FUCAGP levels will change accordingly to the following prognostic status consisting with with CEA levels. The patient L who underwent the first and second line chemotherapy treatments after operation also survived during the period of observation up to 1641 POD. The FUCAGP levels retained at the normal range for over 4 years after operation and CT scans conducting 8 times periodically indicated no recurrence or metastasis of tumors occurred in the patient (data not shown). The patient N who underwent the first line chemotherapy treatment showed strikingly low levels of FUCAGP and no positive results obtained from CT scans during the period of observation even though AGP concentrations and CEA in the same serum samples retained over the cut-off levels. Therefore, FUCAGP levels of patients with good prognosis and survived during the follow-up periods found to retain around the cut-off level in response to the latest line treatment of chemotherapy. Whereas, as shown in patient O (Fig 4b), FUCAGP levels in three patients (patients O, P, and Q in Table 1) who had not received chemotherapy during the period of observation showed to retain around the cut-off level after operation. These patients had no special remarks nor positive results from CT scans or diagnostic tumor markers. Therefore, FUCAGP levels in patients who survived during the period of observation without occurrence of recurrence or metastasis of tumors seemed to retain as low as the healthy controls. AGP concentrations changed differently from FUCAGP levels and no correlation was seen with their levels at all. A sudden increase of AGP concentrations in serum occurred in association with surgical intervention was also seen in all the patients with good prognosis followed by falling the level shortly. Concentrations of serum AGP then seemed to be maintained at relatively low levels except when transient elevation occurred depending on their clinical events.

### Suppressed expression of fucosylated glycans in AGP

**Fig 5:**
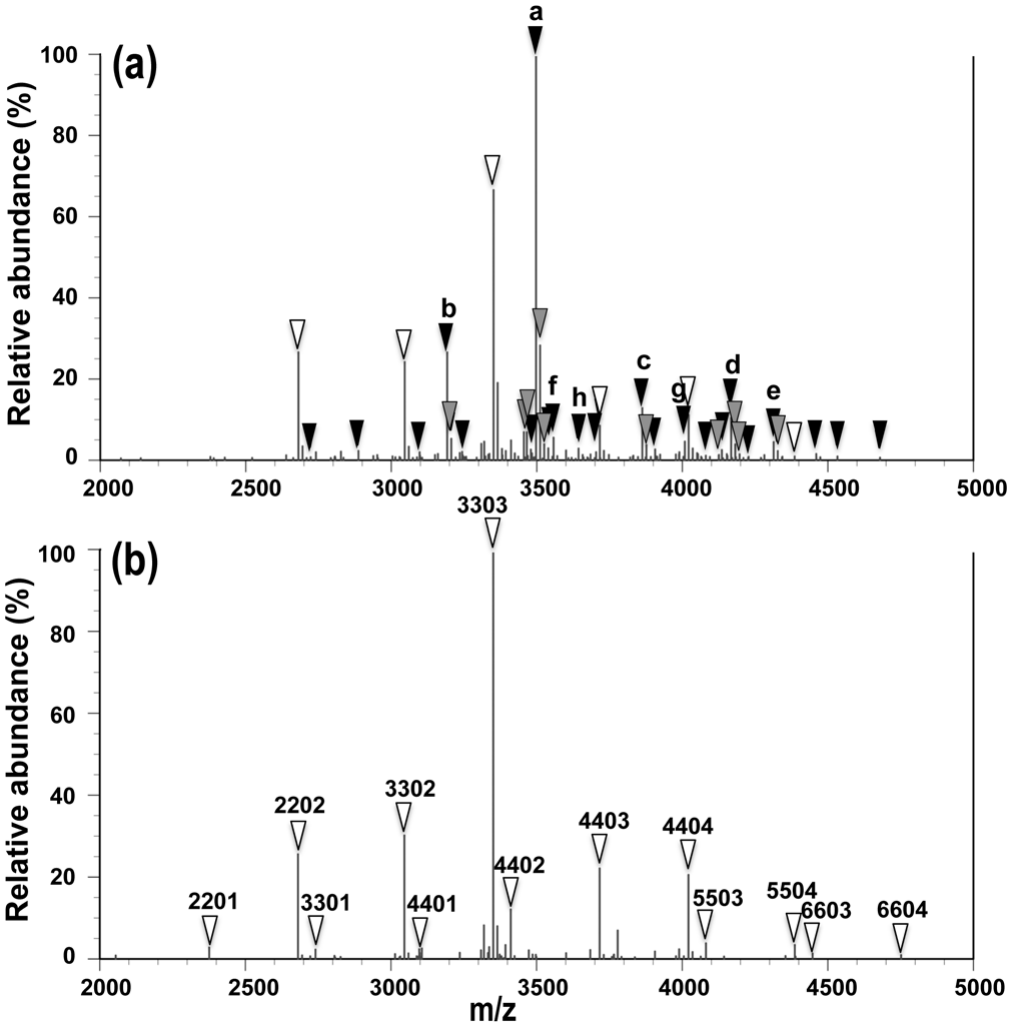
Mass spectra of AGP glycans isolated from a FUT6-deficient patient and a patient with a poor prognosis. Mass spectra obtained from a patient with a poor prognosis (a) (patient M in Table 1) and the patient N (b). White triangles indicate defucosylated glycans and black ones indicate fucosylated tri- and tetraantennary glycans assigned. Gray triangles show major subpeaks of methylated forms of fucosylated glycans derived from methyl esterification of sialylated, fucosylated glycans [35] assigned in the spectra. Four-digit (FD) numbers mean the number of each residue in order of [Gal], [GlcNAc], [Fuc] and [NeuAc] consisting of every glycan chains of five ones per one molecule of AGP except the common core structure. Accordingly, 3303 indicates [Gal]_3_[GlcNAc]_3_[Fuc]_0_[NeuAc]_3_ + [Man]_3_[GlcNAc]_2_ [35]. FD numbers of major fucosylated glycans assigned in (a) were as follows; 3313 (a), 3312 (b), 4413 (c), 4414 (d), 4424 (e), 4412 (f), 4423 (g) and 3323 (h). See the details in Text.

As shown above, the patient N had extremely low levels of FUCAGP during the period of observation even though serum AGP concentrations showed over the cut-off level. Fucosylated glycans in AGP were scarcely detected by mass spectrometric analysis of glycans during the period even though afuco-tri- and tetraantennary glycans such as [Gal]_3_[GlcNAc]_3_[NeuAc]_3_ (3303), [Gal]_3_[GlcNAc]_3_ [NeuAc]_2_ (3302), [Gal]_3_[GlcNAc]_3_[NeuAc]_1_ (3301), [Gal]_4_[GlcNAc]_4_[NeuAc]_3_ (4403), [Gal]_4_[GlcNAc]_4_[NeuAc]_4_ (4404), [Gal]_4_[GlcNAc]_4_[NeuAc]_2_ (4402) and [Gal]_4_[GlcNAc]_4_ [NeuAc]_1_ (4401) were assigned (Fig 5b). These glycans are commonly present in AGP glycans and frequently fucosylated in cancer patients as shown in Fig 5a. The patient was, therefore, suggested to possess the lethal mutation of the *FUT6* gene and to lack serum α1,3fucosyltransferase activity [38]. In fact, the patient was determined to possess the *pf* gene homozygously (*pf*/*pf*) indicating occurrence of lethal mutation in the *FUT6* gens which resulted in the absence of fucosylated glycans in AGP [37]. However, a couple of fucosylated glycans were still detected as less than several % of the total glycans in AGP (Fig 4b). Accordingly, it remains to be examined a significance of poorly expressed fucosylated glycans in such a cancer patient with FUT6-deficiency (details of molecular and enzymatic studies on fucosylation of AGP in patient N with FUT6-deficiency will be published elsewhere).

## Discussion

Proteins are frequently modified post-translationally and more than half of all proteins in nature are glycosylated [39] resulting in being present as glycoproteins with covalently attached glycans having highly heterogeneous and diverse structures. It must therefore be plausible that these glycans have significant implication and responsibility for the structure and function of such glycoproteins. It has been widely recognized that glycans play important roles in not only protein folding and clearance but also cell to cell interaction, recognition and binding together with frequent occurrences of modifications in glycans themselves [40–42]. With the aid of detailed analyses of glycan structures in well characterized glycoproteins, it has been demonstrated that aberrant and novel glycans are synthesized associated with various biological processes, in particular, malignancies [2,43]. Hence, cancer-associated changes of glycan molecules in glycoproteins as well as in other glycoconjugates such as glycolipids [44,45] have been widely demonstrated. Further, such changes are recognized as one of the most promising targets to improve existing tumor markers by means of predicting prognosis of cancer patients more precisely [46].

Whereas, since all these glycans are synthesized enzymatically through catalytic actions of a series of glycosyltransferases, such enzymes could be an important target to detect glycan-based changes in malignancies involving possible establishment of a novel biomarker for diagnosis of cancer. In fact, our previous studies demonstrated the usefulness of analyzing α1,2- [47–49], α1,3- [20,21,37,38,50–54], α1,4- [55–58] and α1,6fucosyltransferase [59] activities using specific sugar-acceptors, which emphasized the specific role of the fucosylated antigens appeared to play on carcinogenesis [1,47–49]. In particular, significantly elevated activities of α1,3fucosyltransferase in plasma and/or serum have been observed in patients with diverse cancers [20,21,51–54]. It has, therefore, been suggested that measurement of serum α1,3fucosyltransferase activity has some superiority over diagnosis with tumor-associated antigen levels in the same samples as a novel biomarker for patients in earlier clinical stages and with smaller size of tumors. Although the possibility cannot be excluded that the enzyme with elevated activities is originated from tumor itself as observed in patients with colorectal carcinoma [60], the origin of α1,3fucosyltransferase in serum has been considered to be the liver. It has also been determined that α1,3fucosyltransferase in serum is encoded by the *FUT6* gene and the missense mutation that occurs in the *FUT6* gene results in the deficiency of serum α1,3fucosyltransferase activity [37,38,61]. Therefore, tumor-associated elevation of serum α1,3fuclsyltransferase activities is assumed to occur in the liver but not originated from tumors formed.

Human AGP is generally recognized as the major serum protein with highly glycosylated glycans and to be originated from the liver. Glycans in AGP consist of five complex type glycans including di-, tri- and tetraantennary structures [25,33]. It has also been determined by our recent mass spectrometric analysis that almost all the terminal galactose residues in every glycan chains are sialylated and the α1,3fucosyl residues are present on the lactosamine structures in both tri- and tetraantennary glycans and elongated tetraantennary ones with repeating lactosamine structure. Hence, our previous investigations convincingly demonstrated both presence of a significant correlation between activities of α1,3fucosyltransferase and total amounts of fucosylated glycans in AGP using the same serum samples [34] and absence of fucosylated glycans in AGP obtained from FUT6-deficent individuals [37]. Furthermore, such afuco-glycans of AGP obtained from FUT6-deficient individuals could be fucosylated on their tri- and tetraantennary structures using the recombinant *FUT6* enzyme or purified plasma α1,3fucosyltransferase under the presence of GDP-fucose as a sugar-donor [S. Yazawa, unpublished observation]. It could, therefore, be strongly suggested that tumor-dependent, increased α1,3fucosylated glycans in AGP are synthesized by an action of the hepatic *FUT6* gene encoded α1,3fucosyltransferase with highly elevated activities.

There have been many reports indicating that analyses of changes occurred in both glycan structures and concentrations of AGP in serum could be useful for the diagnosis and successful management of various diseases including diverse cancers [31,62–74]. Our previous studies suggested that fucosylation of AGP implied significant potential as a novel biomarker for diagnosis and prognosis of cancer patients, which must be strongly associated with tumor-dependent changes of serum α1,3fucosyltransferase activity. Indeed, a strong correlation of levels between fucosylated AGP and α1,3fucosyltransferase activity in the same serum samples was demonstrated previously. Further, the fucosylation index of AGP glycoforms and the relative abundance of fucosylated glycans in AGP (FUCAGP) determined by means of CAIE method [34] and mass spectrometric analysis [35], respectively, were found, for the first time, to be useful for predicting postoperative cancer patients.

In the present study, we first focused on levels of FUCAGP and serum AGP concentrations in patients with various cancers in different clinical stages. Levels of FUCAGP and serum AGP were analyzed in relation to their clinicopathological features in patients (n = 30) who received surgical interventions and/or various chemotherapy treatments. As demonstrated previously [34,35], significant elevated levels of both FUCAGP and serum AGP concentrations were observed in cancer patients at preoperative periods when compared with those in healthy controls. No significant correlation was found between levels of FUCAGP and AGP in both cancer patients and healthy controls, respectively. Interestingly, a very low level of FUCAGP was found in a patient who underwent neoadjuvant chemotherapy. Whereas, as demonstrated previously [34,35], increases of relative amount of diantennary glycans rather than those of tri- or tetraantennary glycans in AGP occurred primarily along with a new synthesis of AGP shortly after operation. This rapid response seemed to occur commonly in patients at the time of operation, and to involve as a consequence of the acute phase reaction toward surgical treatment. During the periods, fucosylated glycans were scarcely detected in AGP, since as described above, only tri- and tetraantennary glycans in AGP were fucosylated through α1,3linkages.

Previous studies on glycoforms and corresponding glycan structures also indicated that, secondarily, AGP with highly fucosylated and branched glycan chains increased specifically in patients with poor prognosis but not in patients with good prognosis. Further, levels of FUCAGP were suggested to become a clinically relevant biomarker over existing diagnostic tumor markers. However, levels of FUCAGP and AGP followed in our previous studies [34, 35] were determined for a limited duration up to one and a half year after operation, and in particular, validation of the fucosylation index of AGP glycoforms and levels of FUCAGP was scarcely performed for predicting treatment outcome in therapeutic settings. Accordingly, we then focused on analyzing variability of serum FUCAGP in patients who had been followed for several years after operation and had undergone various chemotherapies during the period in connection with patients’ responses to medication and various chemotherapy treatments. Seventeen patients including ten patients who died due to the recurrence and/or metastasis of tumors were followed up by analyzing levels of FUCAGP and AGP concentrations in their serum samples together with general surveillance with CT scans and tumor marker diagnosis.

Results from follow-up studies of FUCAGP levels conducted depending on patients’ prognoses indicated that elevated levels of FUCAGP were retained and no striking drop of the level was observed in patients who seemed not to respond to any repeated treatments of chemotherapy and died with a short survival after operation. Whereas, patients who survived for a longer period (up to 1639 POD) under the therapeutic settings with repeated chemotherapies showed partly different changes of FUCAGP levels. First, patients whose FUCAGP levels were found to extremely elevate shortly after operation and survived for a longer period with repeated chemotherapy seemed to respond to the first line of adjuvant chemotherapy resulting that the level fell and in some cases dropped below the cut-off level. However, either recurrence and/or metastasis or no experience of complete remission occurred, and subsequently they died due to the progress of disease accompanying constantly elevated levels of FUCAGP. Second, patients whose FUCAGP levels had been retained at relatively low and around the cut-off level under receiving repeated chemotherapy treatments showed an incomplete response to the latest line treatment of chemotherapy resulting in progress of disease and constant elevation of FUCAGP levels.

Whereas, patients who had been survived during the observation period with and without chemotherapy treatment showed either clear response to repeated chemotherapy treatments or no clinical recurrence during the period of observation. It was of particular interest that in these patients with good prognosis, elevated levels of FUCAGP once after receiving the previous line treatment of chemotherapy fell around the cut-off level by receiving the latest line treatment of chemotherapy and low levels of FUCAGP after operation were retained around the normal range without adjuvant chemotherapy treatment. Therefore, these follow-up studies of FUCAGP levels in patients with different prognosis suggested that patients with poor prognosis possessed extremely elevated levels in the latest period but patient with good prognosis seemed to retain relatively low levels around the normal range. Further, in patients who seemed to respond well to chemotherapy treatment and survived, elevated levels found in association with the recurrence and/or metastasis of tumors dropped to the normal range after treatment with the latest line treatment of chemotherapy against such clinical events. It is more noteworthy that all such changes in FCUAGP levels seemed to occur frequently in advance before detection of recurrence and/or metastasis of tumors and respective tumor markers coming up positive.

One of the obvious evidences to provide that fucosylation of the AGP molecule is accompanied solely by the *FUT6* gene-encoded hepatic α1,3fucosyltransferase has been obtained from molecular and enzymatic studies on FUT6 deficient individuals. Lack of serum α1,3fucosyltransferase due to the FUT6-deficiency has scarcely been found so far and only restricted numbers of individuals whose AGP molecule has no fucosylated glycan have been reported previously [37,38,75]. The patient N was supposed to possess the lethal mutation in the *FUT6* genes resulting in possessing the mutant *pf* gene homozygously as demonstrated previously [37]. While, mass spectrometric analysis of AGP glycans in this patient showed that up to several % of glycans in AGP were still fucosylated and a couple of fucosylated glycans were constantly detected during the period of observation (Fig 4b). One of the seven individuals with FUT6-deficiency in our previous study showed to possess a small amount of fucosylated glycoforms in AGP [37]. These fucosylated glycans might be synthesized by the action of a certain α1,3fucosyltransferase other than *FUT6*-encoded α1,3fucosyltransferase. CEA levels in the patient N were continuously above the normal range even though CT scans revealed no significant remarks during the period. Accordingly, CEA levels in this patient might be subjected to be false-positive range as demonstrated recently [76]. It has been widely recognized that CA 19–9 could not be used as tumor marker in Lewis-negative cancer patients because individuals with Lewis negative genotypes lack the key enzyme to synthesize Fucα1,4GlcNAc linkage, part of its antigenic determinant [57,77]. Hence, the results suggest that FUCAGP level could not be useful in patients with FUT6 deficiency as a clinically relevant biomarker.

The function of plasma AGP and its potential physiologic significance as an acute phase protein have generated profound interest [33,78–81]. Generally, AGP has highly glycosylated *N*-glycan chains on the molecule with more than 45% content and with abundant heterogeneity. It has therefore been demonstrated that despite expression of different genomic variants and mRNA levels of AGP, drastic changes occur frequently in not only serum concentrations but also their glycan structures including branching, sialylation and, in particular, fucosylation degrees as posttranslational modification involving in rapid responses to various biological phenomena [25–35,78–84]. It was also shown that each AGP glycoform was renewed at a rate of 15% per day of the plasma pool [85]. A wide variety of changes and a large amount of replacements were also observed in AGP glycans from various cancer patients who received surgical interventions and chemotherapies [34].

Glycoproteomic approach to investigate tumor-associated alteration of glycans in glycoproteins has been developed recently. As one of the most powerful tools, mass spectrometric analyses through MALDI TOF or LC-MS/MS together with specific enrichment methods such as the use of lectin-affinity toward glycans targeted have been applied for a comprehensive analysis of large amounts of glycans. Indeed, findings of usefulness of *Aleuria aurantia* lectin (AAL) for isolation of fucosylated antigens [86,87] and characterization of its detailed binding specificity [88] have been carried on into further applications of this lectin to recent glycoproteomic studies using human plasma and/or serum samples [34,71,73,89–92]. Whereas, AGP is a major serum protein and due to its original aspect as an acute phase protein, changes of AGP concentrations in serum waver depending on the degree of inflammation caused by a series of diseases and biological events. As expected from our previous studies [34,35], serum AGP levels were found to change differently from FUCAGP ones during the follow-up period in most of the patients. It was also suggested that AGP level changed indistinguishably in patients with poor and good prognosis after operation. Further, it was not unusual observation to see aberrantly elevated AGP levels in serum samples from cancer patients up to several times higher than those in healthy controls. It should therefore be important for targeting serum AGP molecule to focus on not only the fucosylated AGP glycans but also relative abundance of fucosylated AGP glycans after exclusion of large amounts of background noise.

In conclusion, in the present study, FUCAGP could be a clinically relevant biomarker of cancer progression as well as cancer prognosis. The FUCAGP level was found to closely relate to patients’ response to various chemotherapies. Further, instead of our previous CAIE technique for measuring AGP glycoforms which was not simple or convenient for assaying large numbers of samples at attempt, the evaluation of AGP glycans in the present study, by means of an AGP-prep-DOCK and mass spectrometric analyses, was aimed for a quick determination of serum AGP glycans. Additionally, recently we developed and improved AGPAS software for very rapid determination of FUCAGP levels in serum samples. These reflect our long standing view that rapid and accurate measurement of FUCAGP in serum samples might advance diagnosis of better treatment outcomes of cancer patients. We believe that changes in FUCAGP level in a cohort of patients followed up while undergoing various chemotherapeutic regimens warrant further investigation.
